# A proposed classification of incisional hernias after kidney transplantation

**DOI:** 10.1007/s00330-025-11841-5

**Published:** 2025-07-31

**Authors:** Kristoffer Huitfeldt Sola, Torkel B. Brismar, Tomas Lorant, Klaus Lange, Erik Rollvén, Antonios Tzortzakakis, Yi-hua Zhang, Ulf Fränneby, Helena Genberg

**Affiliations:** 1 Unit of Radiology, CLINTEC, Karolinska Institutet, Solna, Sweden; 2 Department of Radiology, Karolinska University Hospital, Solna, Sweden; 3 Section of Transplantation Surgery, Department of Surgical Sciences, Uppsala University, Uppsala, Sweden; 4 Department of Radiology, Danderyd University Hospital, Danderyd, Sweden; 5 Nuclear Medicine and Hospital Physics, Karolinska University Hospital, Solna, Sweden; 6 Department of Surgery, CLINTEC, Karolinska University Hospital, Solna, Sweden; 7 Department of Transplantation Surgery, Karolinska University Hospital, Solna, Sweden

**Keywords:** Computed X-ray tomography, Abdomen, Hernia, Abdominal, Abdominal wall, Diagnostic errors

## Abstract

**Objectives:**

Incisional hernia (IH) following kidney transplantation is underdiagnosed, with reported incidence rates of 1–7%. This study aimed to evaluate IH prevalence using computed tomography (CT), propose a novel classification system correlated with symptomatology, and evaluate its effect on interobserver IH detection rates.

**Materials and methods:**

A retrospective review of adults undergoing kidney-alone transplantation (2010–2017) at two Swedish centres was conducted. Patients with previous ipsilateral transplantation or poor-quality CT were excluded. Abdominal CT scans obtained ≥ 30 days postoperatively were reviewed by a multidisciplinary team (radiologist, transplant surgeon, and hernia specialist, all with > 20 years of experience), blinded to clinical data. Four subtypes of IHs after kidney transplantation were identified: 1A (normal contour, incomplete hernia), 1B (normal contour, complete hernia), 2A (abnormal contour, incomplete hernia), and 2B (abnormal contour, complete hernia). Symptomatology was assessed via medical records. Four external radiologists evaluated the classification’s impact on detection rates.

**Results:**

Of 673 participants, 361 (54%) had evaluable CT scans. IHs were detected in 243 (68%), of which 36% were symptomatic. The proposed classification improved detection rates from 54% to 76% (*p* = 0.03) after an educational intervention. Symptom prevalence increased with hernia severity: 11% in type 1A, 13% in type 1B, 36% in type 2A, and 86% in type 2B (*p* < 0.01). Only 55% of symptomatic participants referred for IH mapping were correctly diagnosed prior to study reassessment.

**Conclusion:**

IHs are prevalent and underdiagnosed after kidney transplantation. The proposed classification improves diagnostic accuracy, correlates with symptomatology, and facilitates clinical management and research.

**Key Points:**

***Question*** *The lack of a generally accepted definition of lateral abdominal wall defects (IH) results in a low detection rate after kidney transplantation*.

***Findings*** *The low detection rate of lateral abdominal wall defects (IH) improved significantly after self-study of the new classification system presented here*.

***Clinical relevance*** *Accurate detection of lateral abdominal wall defects will facilitate surgical repair and improvement of surgical suture techniques*.

**Graphical Abstract:**

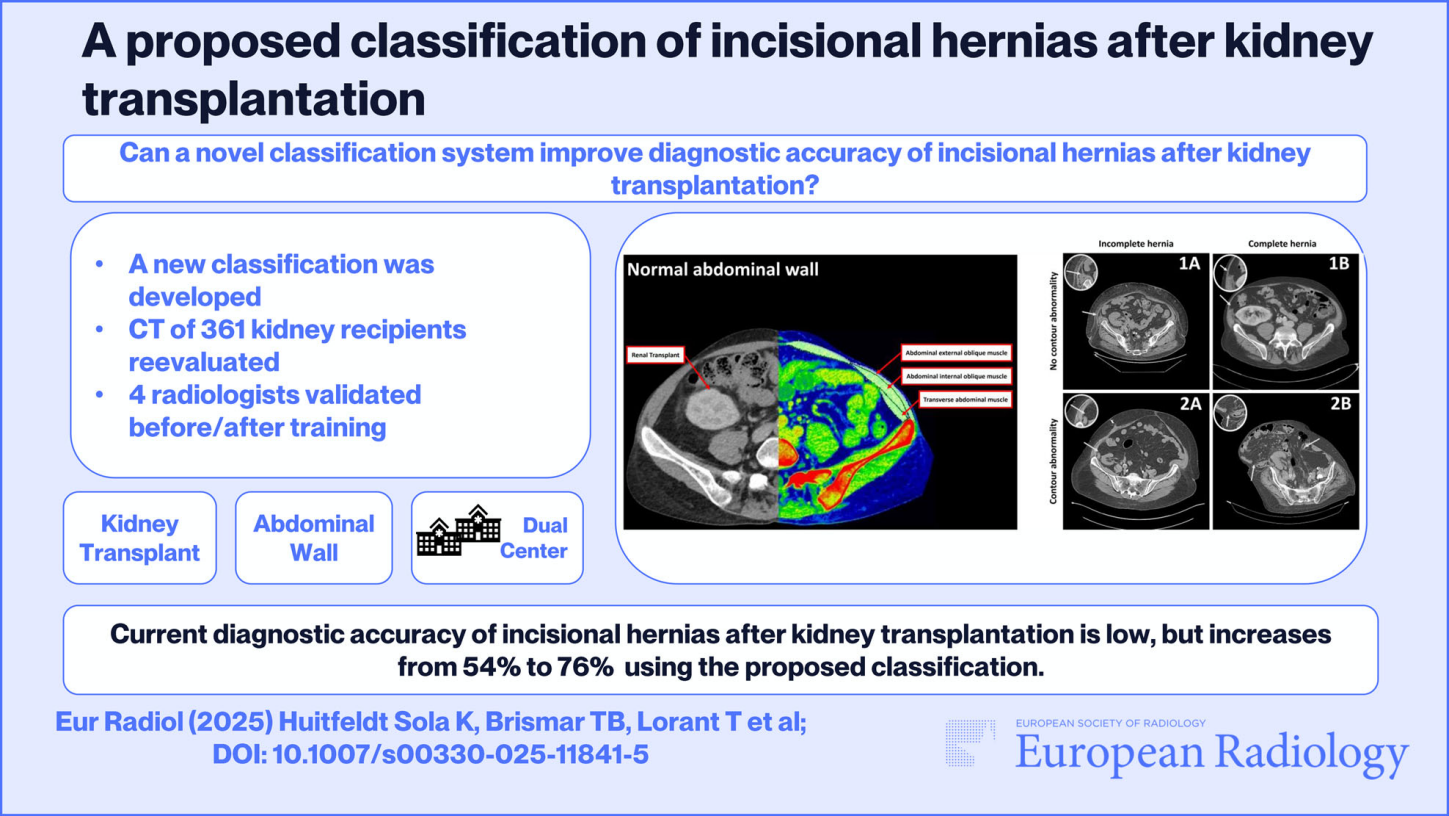

## Introduction

Incisional hernias (IH) are a common complication following kidney transplantation, often leading to severe morbidity, including chronic pain, discomfort, cosmetic issues, and potentially life-threatening complications such as bowel incarceration or strangulation, thereby significantly reducing quality of life [1]. The unique anatomical site of transplant and the immunosuppressed status of recipients pose distinct challenges for IH management [2]. A clear and specific classification system for IH in this population is crucial for accurate diagnosis, standardised reporting, appropriate treatment planning and for facilitating robust research into preventative strategies and optimal repair techniques.

According to the European Hernia Society (EHS, an organisation dedicated to improving hernia care through research and education), an IH is defined as *“Any abdominal wall gap with or without bulge in the area of a postoperative scar perceptible or palpable by clinical examination or imaging”* [3, 4]. While a hernia is often described as a protrusion of tissue or an organ through the abdominal wall, this is a consequence of the underlying abdominal wall gap. The reported incidence of IH after kidney transplantation varies between 1% and 7%, but the condition may be frequently missed, partly due to a lack of a universally accepted definition and partly due to limited awareness of IH after kidney transplantation [5]. This limited awareness may arise from the emphasis on more immediate post-transplant complications, such as graft rejection or infection, combined with the often-subtle presentation of IH in certain patients, especially those with comorbidities that either obscure the symptoms or shift clinical focus elsewhere. Additionally, although IHs often develop during the first months after surgery, some emerge after many years, further increasing the diagnostic challenges [6, 7].

Furthermore, while surgical access for most abdominal procedures is attained through midline incision, abdominal access in kidney transplantation is usually achieved via an extraperitoneal oblique lateral incision, extending from 3 cm above the pubic symphysis to 3 cm medial to the anterior superior iliac spine, commonly referred to as the Gibson incision [8]. Computed tomography (CT) imaging of the abdominal wall is an excellent tool to detect IHs [9]. However, there is considerable interobserver variability in diagnostic accuracy of lateral abdominal wall hernias, which may arise from differences in radiologists’ experience, the subtle presentation, and the lack of a standardised classification system for IH in this specific patient population [10]. Surgery is the only curative treatment for IH [8]. Therefore, an accurate radiologic diagnosis is essential for determining the appropriate treatment approach.

In previous studies on IH after kidney transplantation, identification of IHs has relied on clinical diagnosis and has been limited to those causing clear symptoms [8, 11, 12]. Kidney transplant recipients represent a unique population due to the specific surgical approach and the high prevalence of comorbidities that may mask or delay IH diagnosis [5, 8]. Lateral abdominal wall impairment, which may affect multiple muscle layers and fascia, poses distinct diagnostic challenges not typically encountered following midline incisions [5].

This study aimed to propose a novel classification system for IHs after kidney transplantation, evaluate its diagnostic accuracy and IH detection rates among different observers compared to existing frameworks, assess its correlation with clinical symptomatology, and determine the prevalence of IH in this patient population using CT.

## Materials and methods

### Study design and population

This retrospective study reviewed hospital records of adults (> 18 years) residing in Region Stockholm and Region Gotland, who received a kidney transplant between January 2010 and December 2017 at Karolinska Hospital Stockholm, Sweden, and adults, residing in Region Uppsala, transplanted during the same period at Uppsala University Hospital, Sweden. All abdominal CT scans obtained for any reason > 30 days after transplantation were retrieved. This temporal cut-off was selected to minimise the inclusion of acute post-operative findings (e.g., haematomas) that could interfere with hernia diagnosis and to ensure that detected fascial defects represent persistent IHs, as these are not expected to resolve spontaneously over time. If multiple scans were obtained, the first scan showing an IH was used. The study period was chosen to have a sufficient follow-up duration to capture late-onset IHs.

### Exclusion criteria

Study participants with previous ipsilateral kidney transplantation (and thus a pre-existing incision at the same site), a multiorgan transplant, a poor quality CT scan, or those deceased < 3 months after transplantation were excluded. CT scans performed after hernioplasty for an IH at the transplant site were also excluded from the prevalence analysis. Pre-transplant CT scans were not systematically reviewed for asymptomatic hernias at the future transplant incision site, and the presence of such a hernia was not an explicit exclusion criterion unless related to a prior ipsilateral transplant surgery.

### CT scan protocol and multidisciplinary evaluation

Abdominal CT scans, obtained for any clinical reason > 30 days post-transplantation, were reviewed. These indications included, but were not limited to, investigation of abdominal symptomatology, assessment for suspected transplant-related complications unrelated to hernia, or mapping for a clinically suspected hernia. They were either unenhanced (= 69% of included patients) or contrast-enhanced imaging (arterial phase = 24%, venous phase = 7%), depending on the clinical indication. The slice thickness most used was 5 mm (71%), followed by 3 mm (25%), although other thicknesses were also used (1 mm = 3%, 4 mm = 0.8%, 2 mm = 0.2%). Scans were acquired from a variety of CT scanners, with the three most common being Siemens SOMATOM Definition Flash (34%), GE Revolution CT (19%) and GE Discovery CT750 HD (16%). The most common CT protocol included a tube voltage of 100 kV (62%). Other tube voltages were 120 kV (33%), 80 kV (2%), and 140 kV (3%). All scans were reviewed in axial, coronal, and sagittal planes to ensure consistent image quality.

The CT images underwent multidisciplinary evaluation by a radiologist, a transplant surgeon and a hernia surgeon specialist, all with > 20 years of experience each and blinded to clinical data. Decisions were made in consensus. This consensus decision by the experienced multidisciplinary team, based on the detailed definitions within our proposed classification (see section “Classification of incisional hernias in the low lateral abdominal wall”), served as the reference standard for IH presence and type for the purposes of this study, against which original radiological reports were compared.

### Classification of IHs in the low lateral abdominal wall

**Fig. 1:**
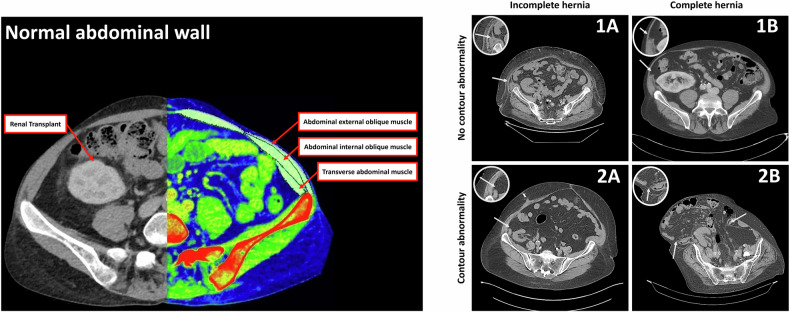
The four subgroups of abdominal IH based on abdominal wall contour and muscle discontinuity. Class 1 hernias exhibit a normal external abdominal wall contour, while Class 2 hernias are characterized by an abnormal contour, such as a visible bulge or protrusion. Muscular discontinuity is graded as A (incomplete: diastasis in either the internal oblique or transverse muscle) or B (complete: diastasis in both the internal oblique and transverse muscles). Class 1A: normal abdominal wall contour with incomplete muscular diastasis (arrow points to diastasis in one deep muscle layer). Class 1B: normal abdominal wall contour with complete muscular diastasis (arrow points to diastasis in both deep muscle layers). Class 2A: abnormal abdominal wall contour (e.g., visible regional protrusion, as may be indicated by altered external anatomical lines) with incomplete muscular diastasis (arrow points to diastasis in one deep muscle layer), with or without visible interruption of the external aponeurosis. Class 2B: abnormal abdominal wall contour (e.g., visible regional protrusion) with complete muscular diastasis (arrows point to diastasis in both deep muscle layers), with or without visible interruption of the external aponeurosis

The classification of IH in the low lateral abdominal wall was developed by the authors (including the aforementioned multidisciplinary core team) to address the anatomical and clinical challenges of IH following kidney transplantation. CT scans first underwent an assessment according to the EHS criteria to confirm the presence of an IH [4]. Subsequently, to provide more specific stratification relevant to lateral hernias in this cohort, IHs were stratified based on the abdominal wall contour into either class 1 or class 2, with class 1 representing normal abdominal wall contour, and class 2 representing abnormal abdominal wall contour, specifically with regional protrusion. Finally, muscular discontinuity was graded; grade A representing a muscular diastasis in either the internal oblique muscle or the transverse muscle, i.e., incomplete hernia, and grade B, representing a muscular diastasis in both the internal oblique muscle and the transverse muscle with or without a visible interruption of the external aponeurosis, i.e., complete hernia. This provided 4 subgroups of IHs: type 1A, 1B, 2A, and 2B (Fig. 1).

### External validation of the proposed classification

**Fig. 2:**
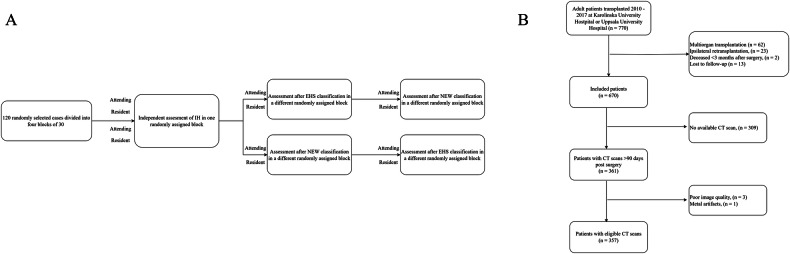
**A** Flow chart of the external evaluation of IH after kidney transplantation by two attending radiologists and two resident radiologists. **B** Flow chart of included study subjects

To subsequently evaluate the potential impact of education on our proposed classification system in improving IH detection rates, four external radiologists (two attending radiologists and two resident radiologists, not participating in the development of the classification) assessed a subset of 120 randomly selected CT scans. The evaluators were each provided with four blocks of 30 CT scans. In the first step, all four evaluators assessed one randomly assigned block. Subsequently, the evaluators were divided into two groups. Each group received a three-slide presentation for self-study on either the EHS hernia classification or the newly proposed IH classification (Supplements 1 and 2), before evaluating another block of 30 study participants. After this evaluation, the groups switched presentations, ensuring that each evaluator had undergone both educational interventions before assessing a final block of 30 participants (Fig. 2A).

### Clinical data collection

Clinical data were obtained retrospectively from the study participants’ medical records. These data were collected primarily to characterise the study cohort and to assess the correlation between hernia types and symptoms. Data included: (age and gender), established risk factors for IH development (body mass index (BMI), smoking history, and diabetes), transplant-related factors (type of renal replacement therapy prior to transplant, donor source), and overall comorbidity burden (Charlson comorbidity index). Specific symptoms of abdominal wall impairment related to the IH, such as localised pain, a palpable or visible bulge, or general discomfort at the surgical site, were extracted from physician notes and patient-reported complaints in the medical records, supplemented by ICD-10 codes indicative of hernia-related issues. Patients were categorised as symptomatic if any of these specific hernia-related complaints were documented in their medical records for the transplant incision site. Consequently, patients were considered asymptomatic if no such symptoms were recorded. The last follow-up date was April 1, 2024.

### Statistical methods

Statistical analyses were conducted using R Core Team (2013) (R Foundation for Statistical Computing). To compare the different subgroups of IHs to the symptomatology of the study participants, and to correlate radiologic misdiagnoses with the proposed classification of IHs, chi-square tests, Fisher’s exact test and Wilcoxon rank-sum tests were performed. Chi-squared tests and *t*-tests were also applied in subgroup analyses to compare categorical and continuous patient characteristics, respectively. A *p*-value of < 0.05 was considered statistically significant.

## Results

### Study participant characteristics

Out of 673 study participants, 361 had abdominal CT scans obtained > 30 days post-surgery available. Four subjects had CT scans of poor image quality and were therefore excluded from the analysis (Fig. 2B). The median age of the study population was 54.2 years (IQR 42–62), the median BMI was 25.7 (IQR 22–27), and 64% were male. Smoking history was present in 51% and 65% had received the kidney from a deceased donor. 70 had diabetes (20%). The median time between transplantation and CT was 50 months (IQR 21–83). The study population characteristics of those with available CT were compared to those without (Table 1). For the 312 participants who did not have a post-transplant CT scan available for review within the study period, no hernia-related diagnoses or symptoms were found. Two cases of emergency reoperation due to incarceration were reported.

**Table 1:** Summary of study population characteristics

Patient factor	Available CT (*n* = 357)	No available CT (*n* = 316)	*p*-value
Age	54.3 (13.2)	48.8 (14.6)	< 0.01
Gender			= 0.05
Male	217 (61%)	216 (68%)	
Female	140 (39%)	100 (32%)	
BMI (kg/m^2^)	25.7 (4.1)	25.1 (3.9)	= 0.07
History of smoking			< 0.01
Yes	199 (56%)	141 (45%)	
No	158 (44%)	172 (55%)	
Type of renal replacement therapy before transplantation			< 0.01
Haemodialysis	221 (62%)	113 (42%)	
Peritoneal dialysis	86 (24%)	100 (32%)	
None	50 (14%)	81 (26%)	
Donor source			< 0.01
Deceased donor	254 (71%)	185 (59%)	
Living donor	103 (29%)	131 (41%)	
Charlson comorbidity index	3.81 (1.52)	3.26 (1.57)	< 0.01
Time between CT and transplantation (months)	54.6 (37.9)	N/A	

Mean value with standard deviation within parentheses is provided. The *p*-value is provided from a *t*-test when numerical data were used, and from a Chi-square test when categorical data were used

### IH prevalence and symptomatology

**Fig. 3:**
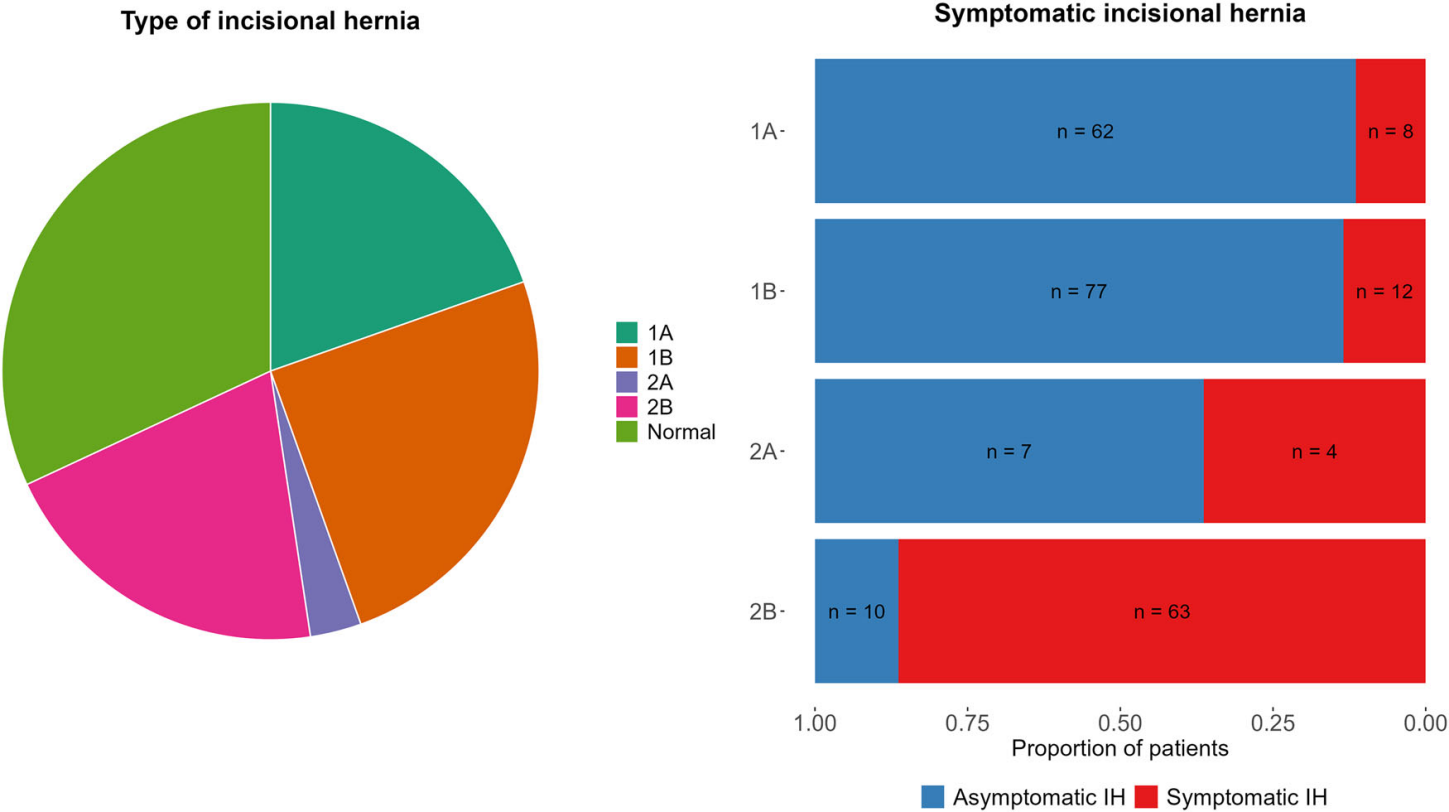
Distribution of IH and the proportion of symptomatic individuals in each class

Radiologic IHs were detected in 243 study participants (68% of subjects with CT scans) (Fig. 3). Out of these, 70 (corresponding to 29% of subjects with posttransplant CT scans) were incomplete hernias with no contour abnormality (type 1A), 89 (corresponding to 37% of subjects with CT) were complete hernias with no contour abnormality (type 1B), 11 (corresponding to 4% of subjects with CT) were incomplete hernias with contour abnormality (type 2A), and 73 (corresponding to 30% of subjects with CT) were complete hernias with contour abnormality (type 2B). The median transversal size of the IHs overall was 61 mL with an interquartile range (IQR) of 37–89 mL. The median transversal size increased with IH severity, being 44 mL for type 1A (IQR 28–61), 52 mL for type 1B (IQR 7–71), 80 mL for type 2A (IQR 51–105) and 105 mL for type 2B (IQR 79–128). Statistical comparisons using the Wilcoxon rank-sum test showed no significant difference in size between IH type 1A and 1B (*p* = 0.64), a significant difference between type 1B and 2A (*p* < 0.01), and no significant difference between type 2A and 2B (*p* = 0.11). However, the difference in size between types 1A/1B and type 2B was significant (*p* < 0.01), reflecting the progression of hernia severity.

The relationship between the time from transplant to CT scan and hernia size was weak, with a correlation coefficient of .04, indicating no significant linear association. However, symptomatic hernias were significantly larger than asymptomatic ones, with a mean hernia size of 91 mm in symptomatic cases compared to 49 mm in asymptomatic cases (*p* < 0.01). Hernioplasty was performed in 32 of the symptomatic patients (37%).

Out of the 243 subjects with IHs, 87 (36%) had hernia-like symptoms mentioned in their medical records. The percentage of symptomatic subjects also increased with IH severity; 8/70 with type 1A (11%), 12/89 with type 1B (13%), 4/11 with type 2A (36%) and 63/73 with type 2B (86%) (Fig. 3). Statistical comparison using Fisher’s exact test showed a significant association between IH classification severity and presence of symptoms (*p* < 0.01).

### Radiologic detection of IHs: original reports vs study reassessment

**Fig. 4:**
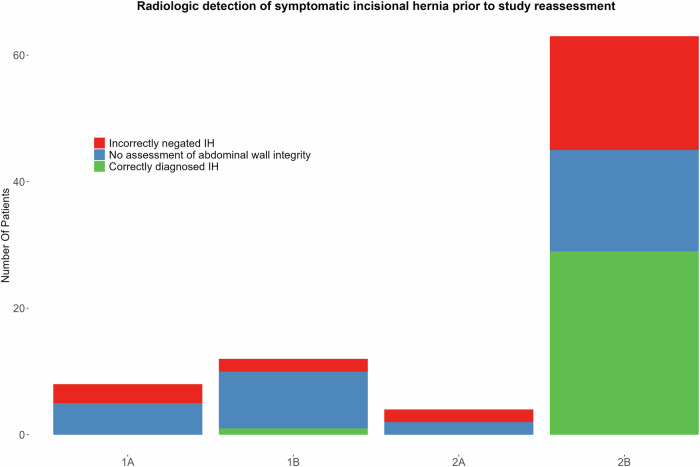
Prevalence of symptomatic IH and radiologic detection of IH prior to study reassessment. Green represents correctly diagnosed IH, red incorrectly negated IH and blue represents no reported assessment of the abdominal wall integrity

Of the 87 participants with hernia-related symptoms, 55 had undergone CT scans specifically requested for IH mapping based on clinical suspicion. Before study reassessment, only 30 of those IHs were radiologically detected and documented (30/55, 55%) (Fig. 4). Of these 30 initially diagnosed IHs, our classification identified all but one as type 2B; the remaining case was type 1B.

The other 25 symptomatic study participants who had CT scans for IH mapping did not have an IH diagnosis in their original radiology reports. However, upon our multidisciplinary team’s re-evaluation using the proposed classification, IHs were identified in all 25 of these cases. These previously unreported IHs were classified as: 3 type 1A, 2 type 1B, 2 type 2A, and 18 type 2B.

**Fig. 5:**
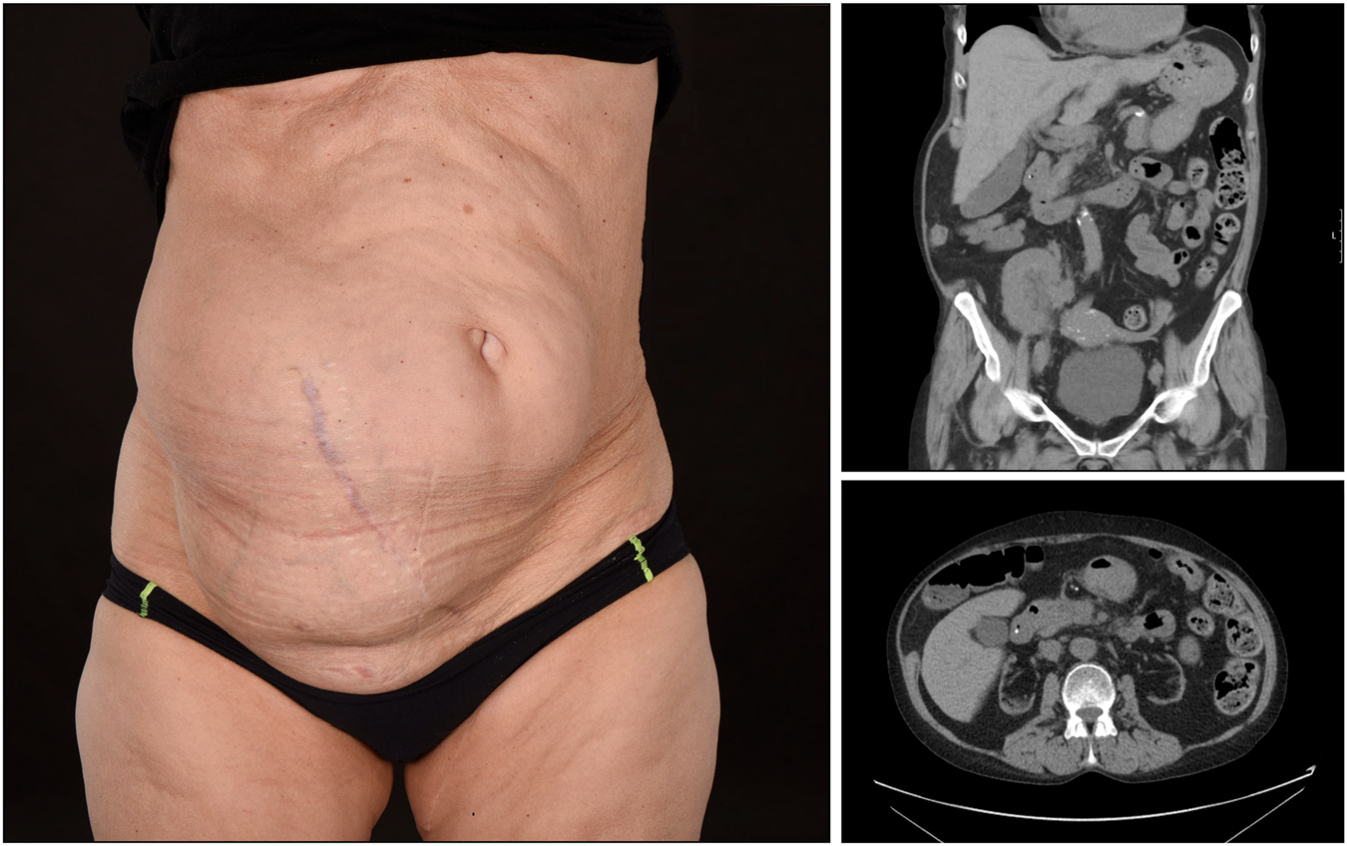
A study participant seeking care for right side protrusion of the lateral abdominal wall after kidney transplantation, the original CT report negating the presence of IH. Using the proposed classification, the IH is graded as a type 2B, 132 mm. The IH was later intraoperatively verified during hernioplasty. The study participant has consented to the use of these images

Furthermore, 32 symptomatic study participants ultimately underwent hernioplasty. Review of their preoperative CT reports showed that an IH was correctly identified prior to surgery in 19 of these 32 patients (59%). In 4 cases, the CT report did not comment on abdominal wall integrity, and in 7 cases, an IH was explicitly denied. Importantly, an IH was intraoperatively verified in all patients who underwent surgery. For the 30 patients who had surgery and whose preoperative CTs were reassessed in our study, all IHs were detectable using our proposed classification; these included 1 type 1A, 2 type 1B, 1 type 2A, and 26 type 2B (Fig. 5).

### Evaluation of the proposed classification

**Fig. 6:**
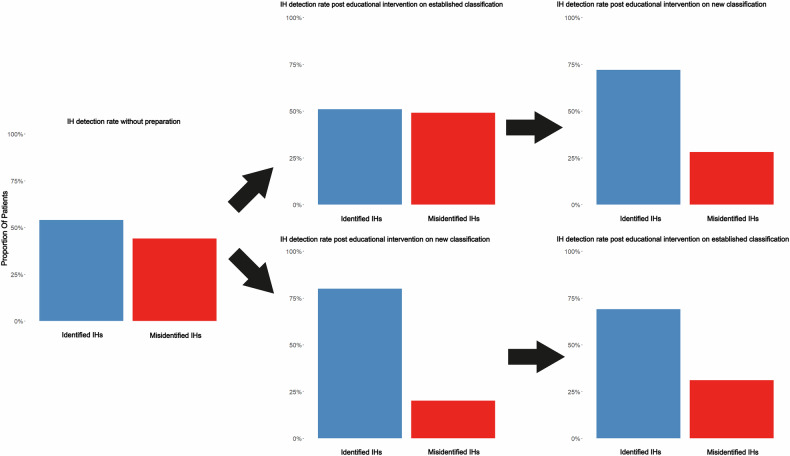
The detection rates of IHs by four external radiologists before and after provision of slide decks on the established EHS hernia classification and our herein proposed classification. Two radiologists started with the EHS classification, and two with our proposed classification. Subsequently, there was a crossover of educational material between the groups

Without any educational intervention for the external evaluators, the average detection rate of IH was 54% (range 44–70%). After the provision of the educational slide presentation on our proposed IH classification, the average detection rate rose from 54% to 76% (range 68–80%, *p* = 0.03). In contrast, the educational slide presentation on the EHS classification had no significant effect on detection rates (*p* = 0.93) (Fig. 6).

## Discussion

The clinical impact of IH in kidney transplant recipients extends beyond mere asymptomatic defects. Symptomatic hernias can cause persistent pain, limit physical activity, negatively affect body image, and carry the risk of acute complications requiring emergency surgery, which is particularly risky in this vulnerable patient group. Therefore, a precise classification correlated with symptoms, like the one proposed, is vital for guiding clinical decision-making.

There is a high likelihood that the incidence of IH after kidney transplantation is heavily underreported. The explanation may be multifactorial, with clinician unawareness being of importance. Many IHs are observable at CT and may also be asymptomatic. Only 36% of the study participants with IH in this study had documented symptoms. However, the size and severity correlated well with the symptomatology, but a small proportion of study participants with large hernias had no symptoms reported. This can possibly be explained by care providers' unawareness and by extensive comorbidity, IH presumably being one of the study participants’ lesser health issues.

In this study, only 12% of study participants with IH detectable by CT had it mentioned in the original radiology report, and only 54% of the symptomatic study participants with a CT scan for IH detection and mapping received a correct diagnosis. A plausible explanation is that the current technique of diagnosing IH on CT, as well as clinically, focuses on finding a herniation with a clear abdominal gap or fascial defect [10, 13].

Our study indicates that IHs are prevalent after open kidney transplantation, with a radiologic detection rate of 68%. This is substantially higher than previously reported rates of 1–7%, which were based on clinical diagnosis alone [5]. The discrepancy likely reflects the underdiagnosis of IH due to limited awareness and the subtle presentation of lateral abdominal wall hernias. The underdiagnosis of type 2 hernias, particularly type 2B, is concerning given their association with significant symptomatology (86% in our study).

However, lateral hernias may have an intact aponeurosis of the external oblique muscle, even when there is a distorted abdominal contour as seen in the Spigelian hernia [14]. This may partially explain why midline IHs have been reported at a higher rate than lateral IHs in previous studies [5, 12]. The anatomical location, with its three layers of muscle and fascia, apparently causes difficulties in both the clinical and the radiologic assessment.

Conventional classifications for IH are broad and nonspecific [3, 4, 15–18], with only a few using location as a parameter [4, 16, 17]. A more specific and preferably simple classification of IH following lateral incision, would be of great value for kidney recipients. In addition, a standardised classification of IH following kidney transplantation could improve comparability between scientific studies. The radiologist's use of the term “abdominal wall diastasis” might be more correct, as there is often no apparent herniation of tissue.

The evaluation of our proposed classification focused on its educational utility; after a brief educational intervention, the average IH detection rate by external radiologists significantly increased from 54% to 76%. The lack of improvement in detection rates after the EHS classification intervention underscores the limitations of conventional systems in diagnosing lateral abdominal wall hernias. The proposed classification, tailored to kidney transplantation, demonstrated significantly higher diagnostic accuracy, suggesting its potential utility in clinical practice and research. This suggests that the structured criteria of our classification can lead to more consistent IH identification. While formal inter-reader reliability was not assessed, the improved detection rates point to its potential to reduce missed diagnoses.

A low detection rate of IHs has several implications. Not only may it impede adequate clinical management, but also complicate research. Prediction models, for example, have been developed to identify risk factors for IH [11, 19, 20]. If the proportion of unidentified IHs in the input data of those models is comparable to what has been observed in our study, there is a risk that these models are hampered by false negative cases, probably resulting in an underestimation of their efficacy.

Our findings indicate that the most significant surgical intervention needs are among patients with type 2B IHs, characterised by contour abnormalities and diastasis of both the internal oblique and transverse abdominal muscles. These individuals are highly symptomatic, 86% reporting hernia-related symptoms, underscoring the clinical relevance of this classification. Furthermore, type 2A defects, although less common, also show a notable association with symptomatology and may eventually require surgical management. In contrast, type 1A and 1B defects, despite their higher prevalence, seemed more often asymptomatic, suggesting that conservative management may be more appropriate for most such cases. While symptoms remain a key driver for surgical intervention, our classification system aims to provide clinicians with a reliable and standardised tool to guide their decision-making and provide radiologists a clear and simple framework for diagnosis.

Limitations of our study include the retrospective design. CT imaging of the abdomen obtained after transplantation was therefore only available for 54% of the entire cohort of kidney recipients, and the follow-up time varied. IHs may emerge months to years after abdominal surgery [21], so a few late emerging IHs might have been overlooked. However, several study participants had multiple CT scans postoperatively, and for all those diagnosed with an IH, the IH was present at the first postoperative CT scan, implying that IHs usually emerge early after surgery. Moreover, we also sourced information about study participants' symptomatology from the participants’ medical records, which could result in a few IHs being missed due to limitations in the follow-up methodology. Furthermore, a systematic review of pre-transplant CT scans, when available, was beyond the scope of this study but could provide information on baseline abdominal wall integrity. Yet, strengths of the study include the multicentre format and the blinded external evaluation of the classification.

In conclusion, our study indicates that IHs are prevalent after open kidney transplantation and are heavily underdiagnosed. Using the proposed classification, the severity of such hernias can be accurately described and correlated with symptomatology. The proposed classification of IH after open kidney transplantation may therefore aid radiologic detection, facilitating both correct diagnosis and adequate management, as well as further research.

## Supplementary information

ELECTRONIC SUPPLEMENTARY MATERIAL

EHS Hernia classification

New classification of lateral abdominal wall hernias

## Data Availability

The data that support the findings of this study are available from the corresponding author upon reasonable request.

## Abbreviations

BMI: Body mass index
CT: Computed tomography
EHS: European Hernia Society
IH: Incisional hernia
IQR: Interquartile range
