# Supplementary material for: A proposed classification of incisional hernias after kidney transplantation
Source: Eur Radiol. 2025 Jul 31;36(2):1483–92. doi: 10.1007/s00330-025-11841-5 (PMC12953296; doi:10.1007/s00330-025-11841-5)
Supplement: Supplementary file 1 — ELECTRONIC SUPPLEMENTARY MATERIAL [file 330_2025_11841_MOESM1_ESM.pdf]

A proposed classification of incisional hernias after kidney transplantation

ELECTRONIC SUPPLEMENTARY MATERIAL

Eur Radiol (2025) Huitfeldt Sola K, Brismar TB, Lorant T, et al.

EHS Hernia classification

PMID: 19495920

Definition of incisional hernia

It was decided to use the definition proposed by Korenkov et al. [4]: "Any abdominal wall gap with or without a bulge in the area of a postoperative scar perceptible or palpable by clinical examination or imaging".

Lateral hernias The borders of the lateral area are defined as (Fig. 2).

1. cranial: the costal margin
2. caudal: the inguinal region
3. medially: the lateral margin of the rectal sheath
4. laterally: the lumbar region.

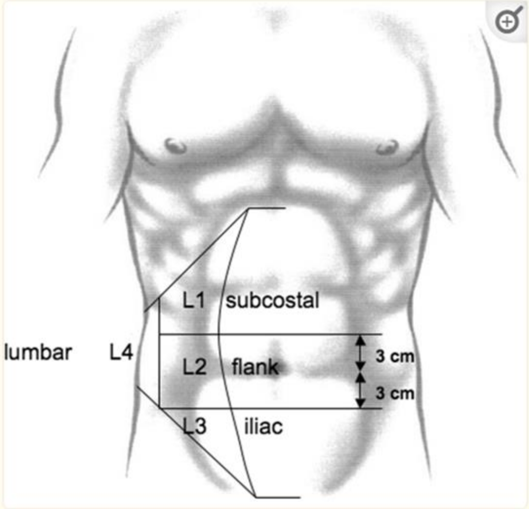

Fig. 2

To classify lateral incisional hernias, four zones lateral of the rectus muscle sheaths were defined

Thus, four L zones on each side are defined as:

1. L1: subcostal (between the costal margin and a horizontal line 3 cm above the umbilicus)
2. L2: flank (lateral to the rectal sheath in the area 3 cm above and below the umbilicus)
3. L3: iliac (between a horizontal line 3 cm below the umbilicus and the inguinal region)
4. L4: lumbar (latero-dorsal of the anterior axillary line)

The width of the hernia defect was defined as the greatest horizontal distance in cm between the lateral margins of the hernia defect on both sides. In case of multiple hernia defects, the width is measured between the most laterally located margins of the most lateral defect on that side (Fig. 3).

9495920

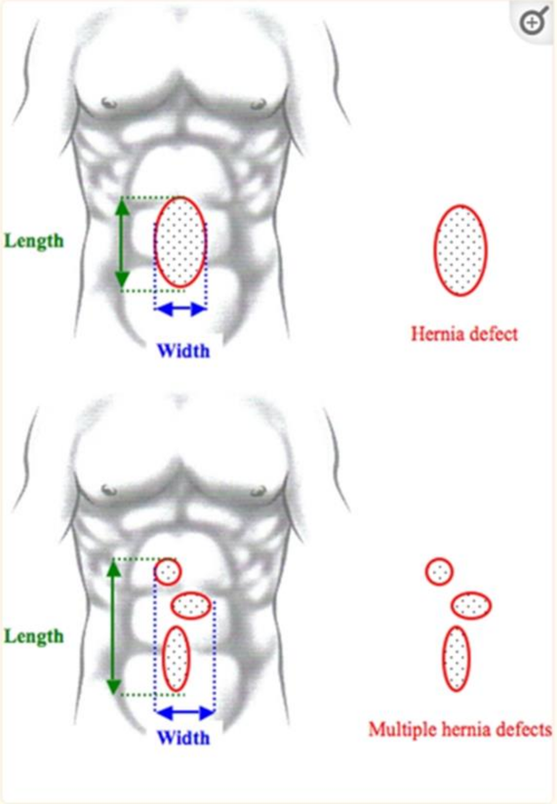

Fig. 3

Definition of the width and the length of incisional hernias for single hernia defects and multiple hernia defects

In summary

- From PMID: [19495920](19495920)

Table 3

European Hernia Society classification for incisional abdominal wall hernias

E H S<br>Incisional Hernia Classification			
Midline	subxiphoidal	M1	
	epigastric	M2	
	umbilical	M3	
	infraumbilical	M4	
	suprapubic	M5	
Lateral	subcostal	L1	
	flank	L2	
	iliac	L3	
	lumbar	L4	
Recurrent incisional hernia?	Yes O	No O	
length: cm		width: cm	
Width cm	W1 <4cm O	W2 ≥4-10cm O	W3 ≥10cm O

Eur Radiol (2025) Huitfeldt Sola K, Brismar TB, Lorant T, et al.

New proposed classification of lateral abdominal wall hernias

Eur Radiol (2025) Huitfeldt Sola K, Brismar TB, Lorant T, et al.

Parameters

- Firstly: Is there an **abdominal wall gap** perceptible or palpable by clinical examination or imaging?
  If YES → Hernia

- Secondly: Is there an abnormality in the abdominal wall outline or a **protrusion of the abdominal wall**?
  If NO → type 1
  If YES → type 2

- Thirdly: Is the gap located in the **transverse abdominal muscle, internal oblique muscle or both**.
  If one layer → class A
  If both layers → class B

4 Types of IH

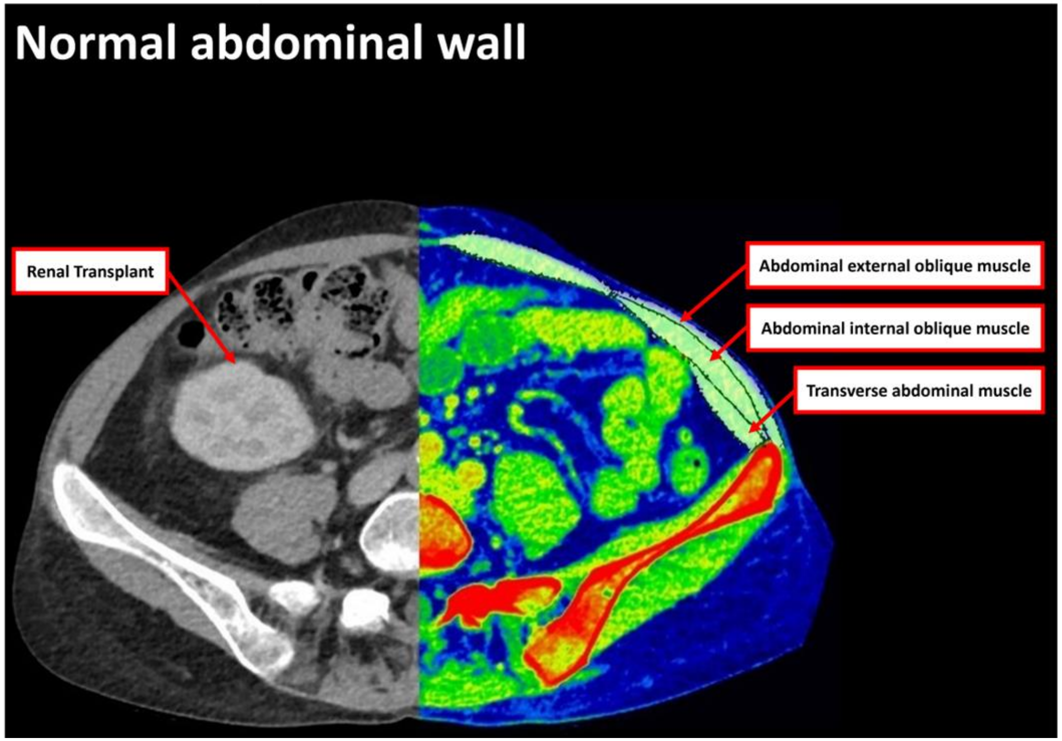

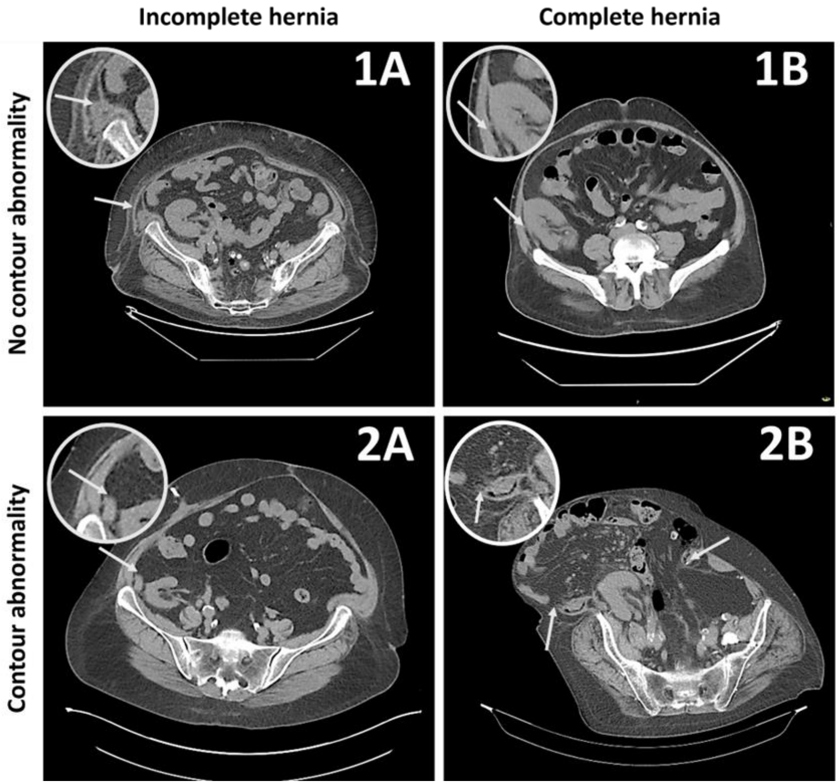

Eur Radiol (2025) Huitfeldt Sola K, Brismar TB, Lorant T, et al.
