# Supplementary material for: A proposed classification of incisional hernias after kidney transplantation
Source: Eur Radiol. 2025 Jul 31;36(2):1483–92. doi: 10.1007/s00330-025-11841-5 (PMC12953296; doi:10.1007/s00330-025-11841-5)
Supplement: Supplementary file 2 — EHS Hernia classification [file 330_2025_11841_MOESM2_ESM.pptx]

## Slide 1
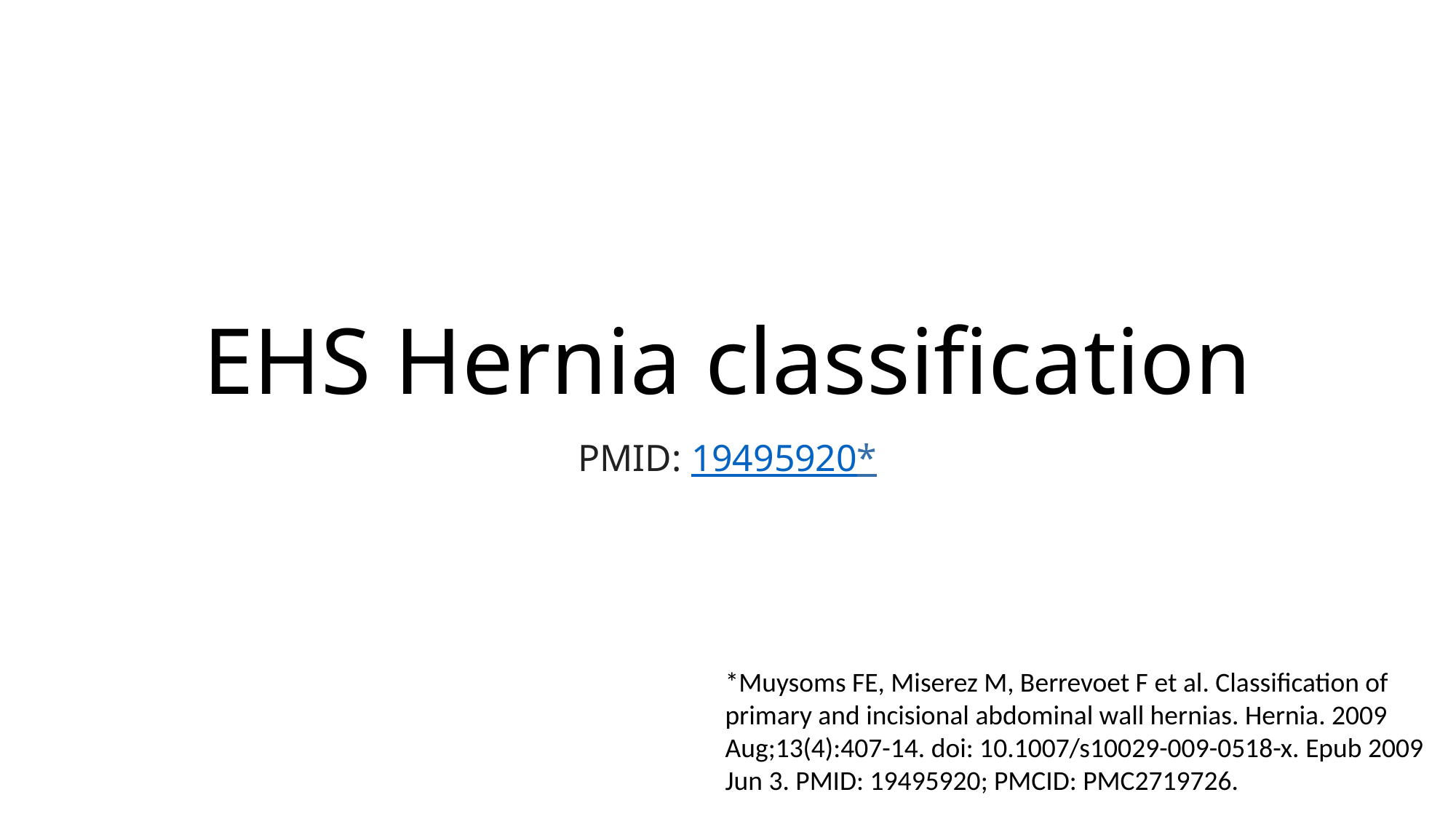

# EHS Hernia classification
PMID: 19495920*
*Muysoms FE, Miserez M, Berrevoet F et al. Classification of primary and incisional abdominal wall hernias. Hernia. 2009 Aug;13(4):407-14. doi: 10.1007/s10029-009-0518-x. Epub 2009 Jun 3. PMID: 19495920; PMCID: PMC2719726.

## Slide 2
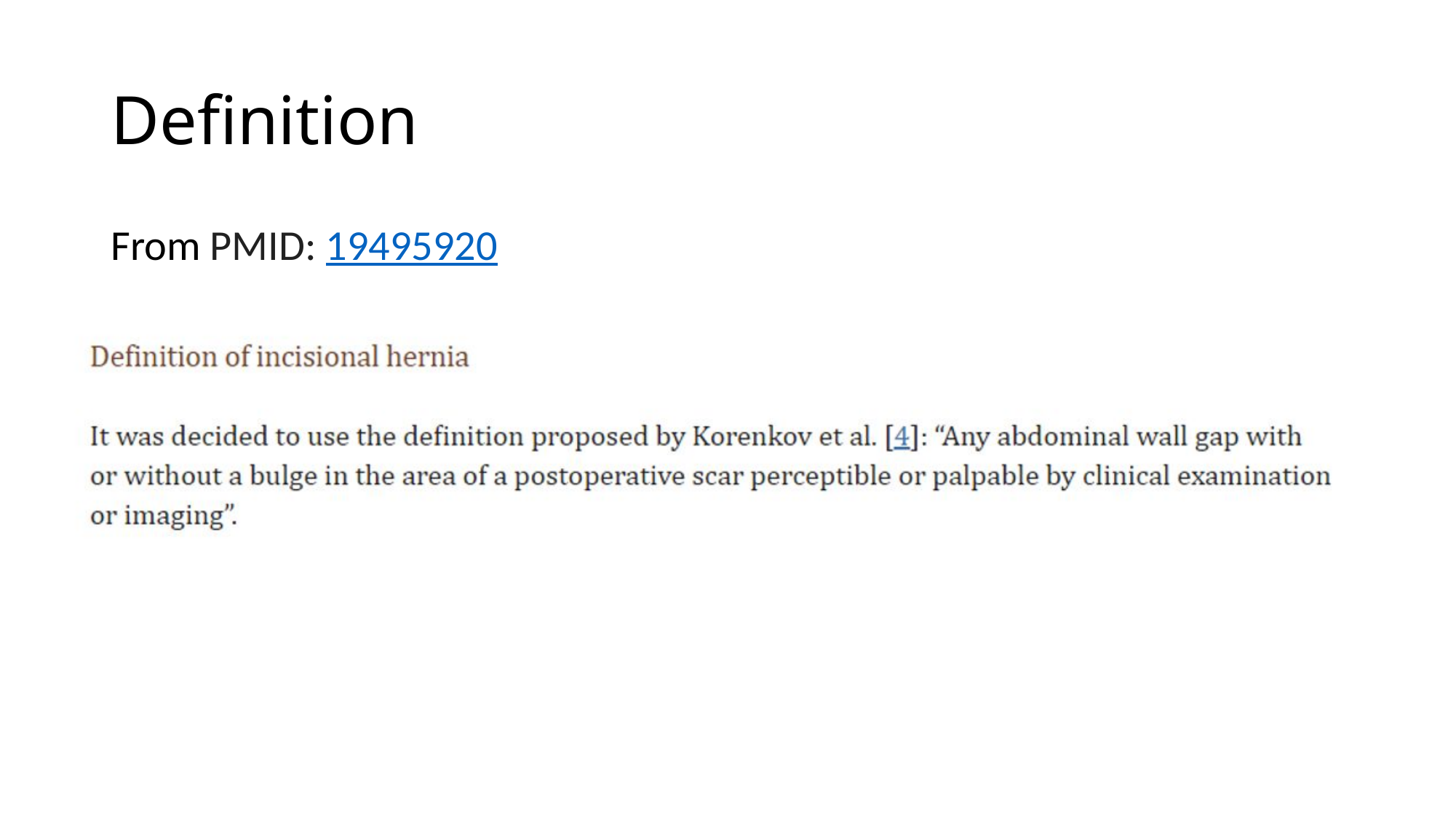

# Definition
From PMID: 19495920

## Slide 3
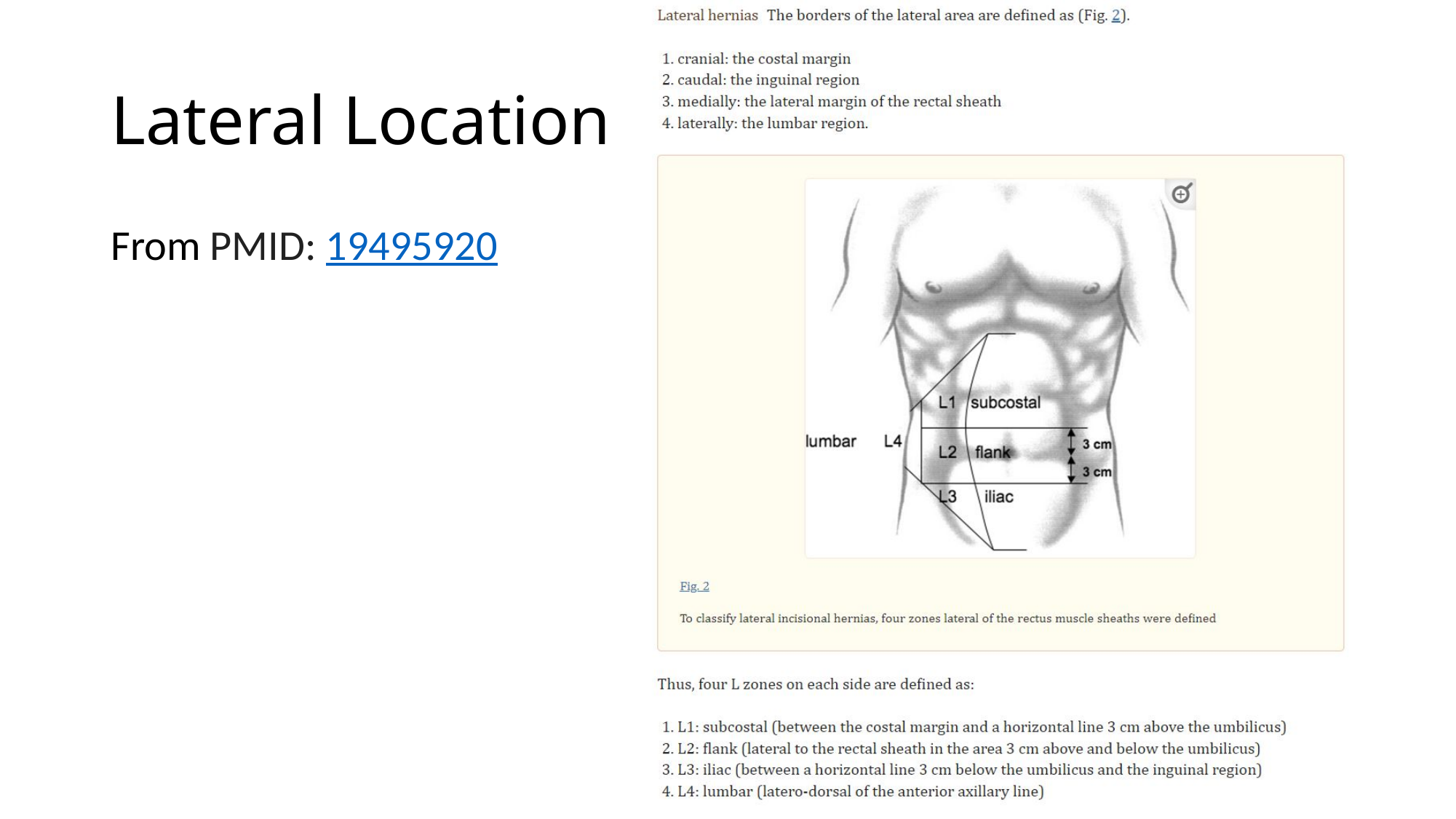

# Lateral Location
From PMID: 19495920

## Slide 4
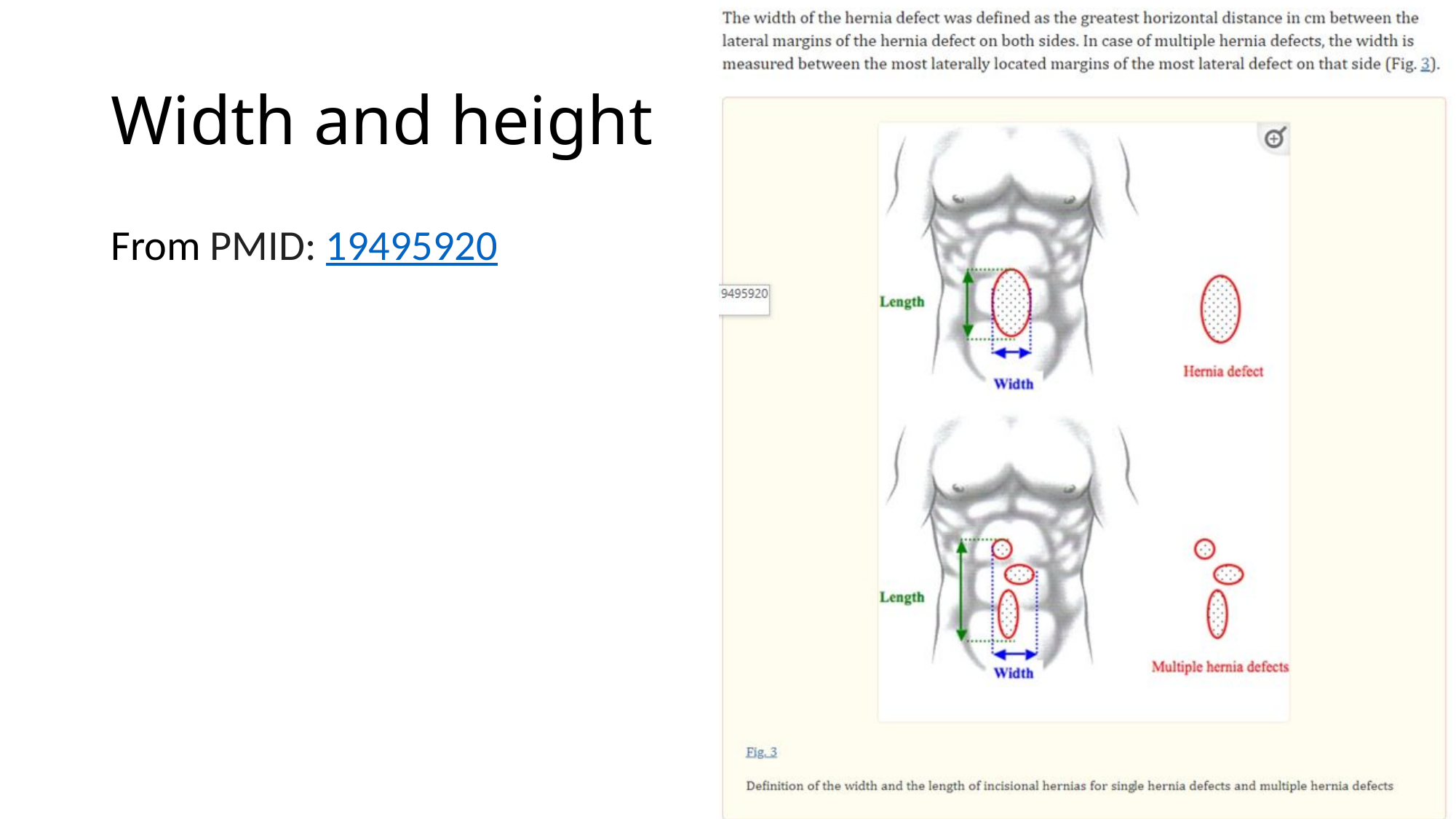

# Width and height
From PMID: 19495920

## Slide 5
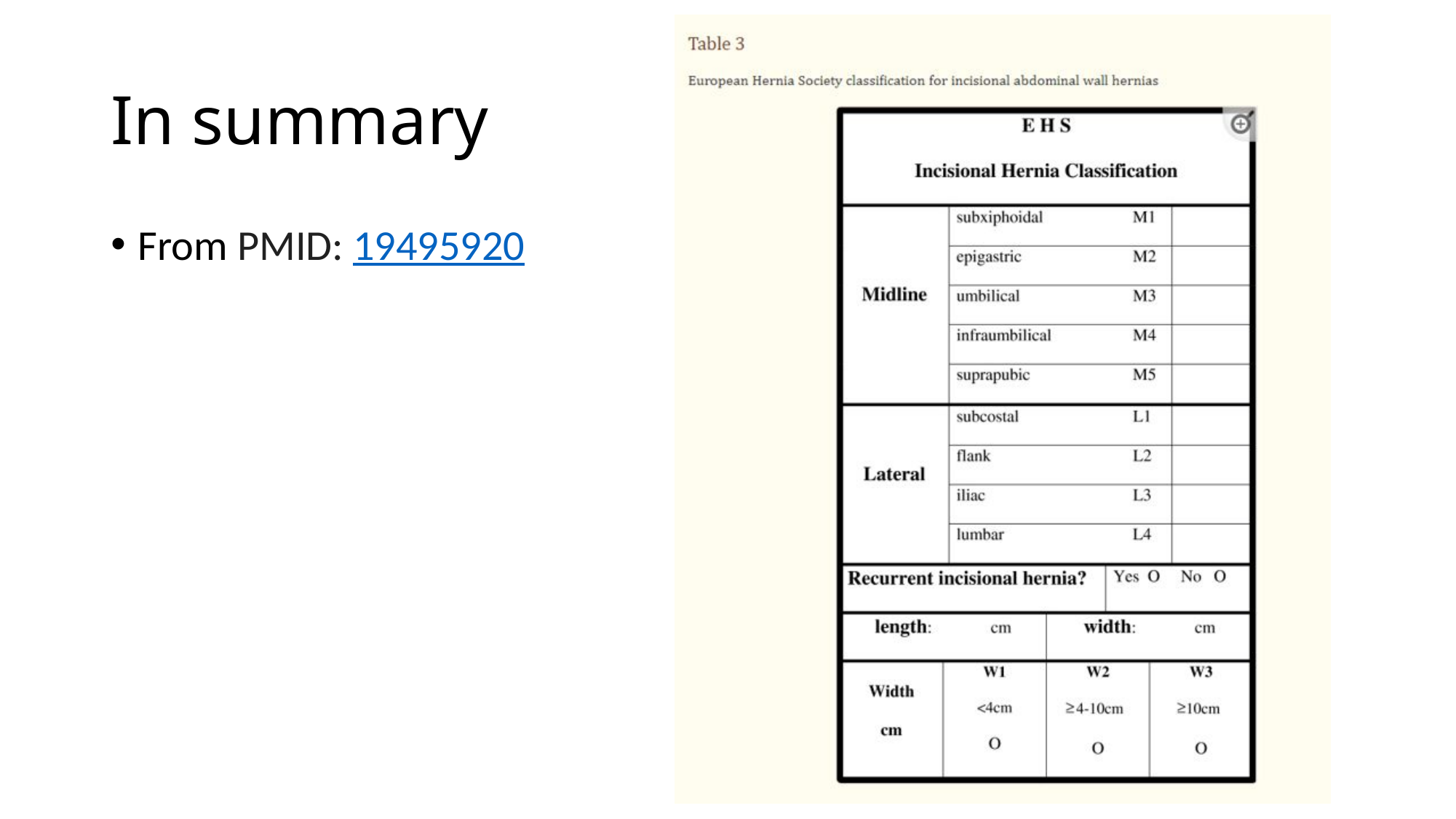

# In summary
From PMID: 19495920
