# Supplementary material for: A proposed classification of incisional hernias after kidney transplantation
Source: Eur Radiol. 2025 Jul 31;36(2):1483–92. doi: 10.1007/s00330-025-11841-5 (PMC12953296; doi:10.1007/s00330-025-11841-5)
Supplement: Supplementary file 3 — New classification of lateral abdominal wall hernias [file 330_2025_11841_MOESM3_ESM.pptx]

## Slide 1
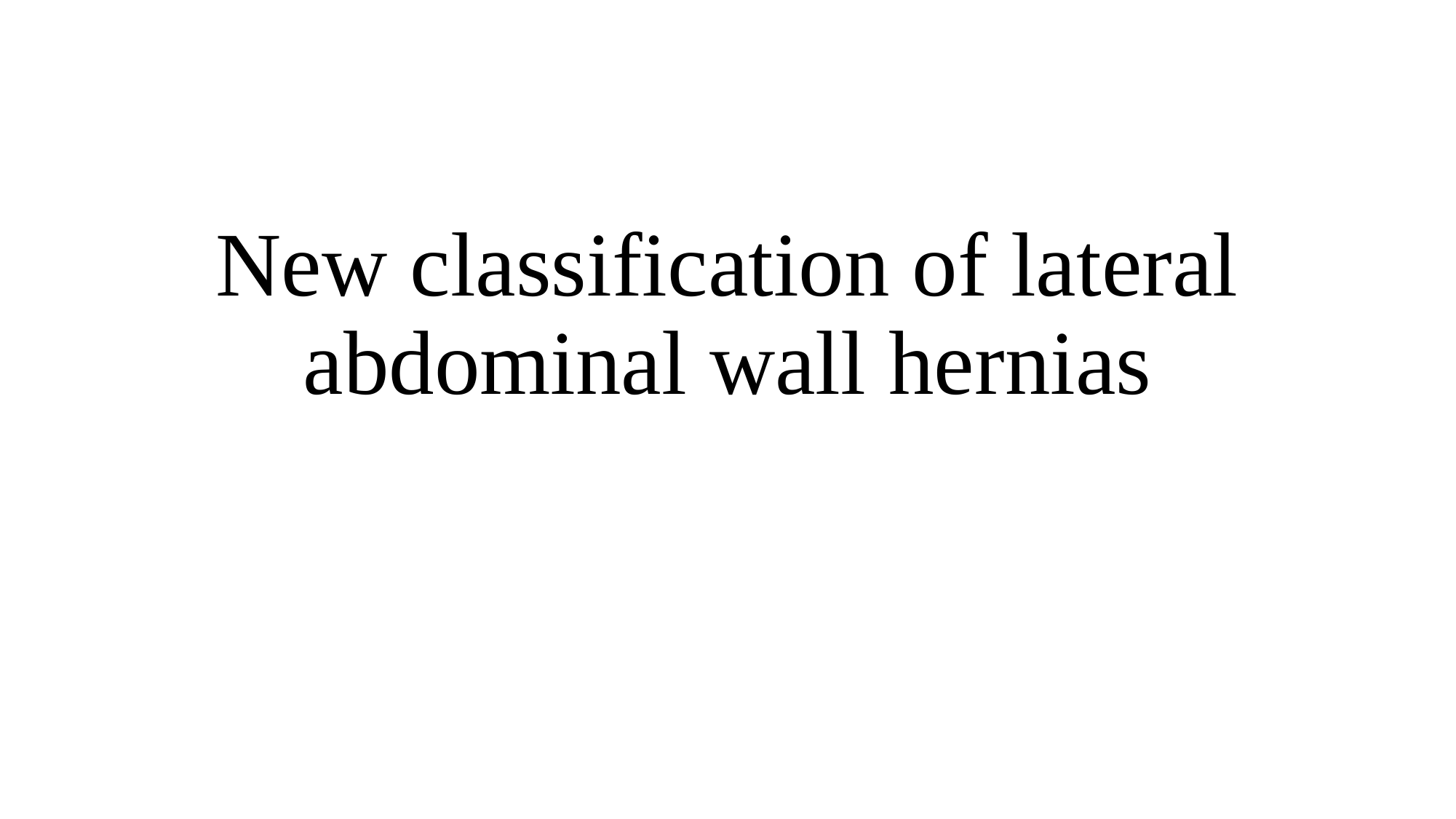

# New classification of lateral abdominal wall hernias

## Slide 2
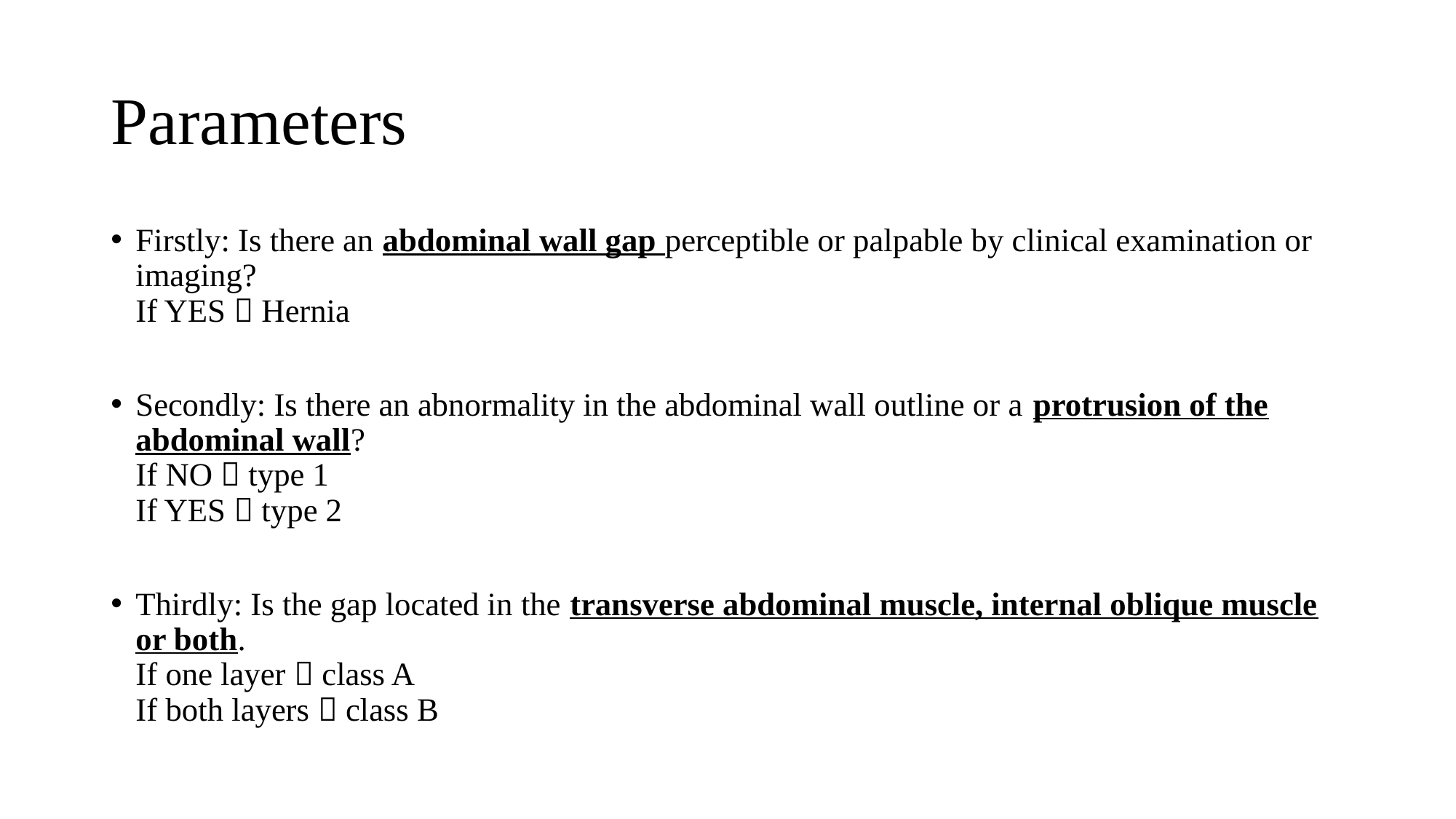

# Parameters
Firstly: Is there an abdominal wall gap perceptible or palpable by clinical examination or imaging?
If YES  Hernia
Secondly: Is there an abnormality in the abdominal wall outline or a protrusion of the abdominal wall?
If NO  type 1
If YES  type 2
Thirdly: Is the gap located in the transverse abdominal muscle, internal oblique muscle or both.
If one layer  class A
If both layers  class B

## Slide 3
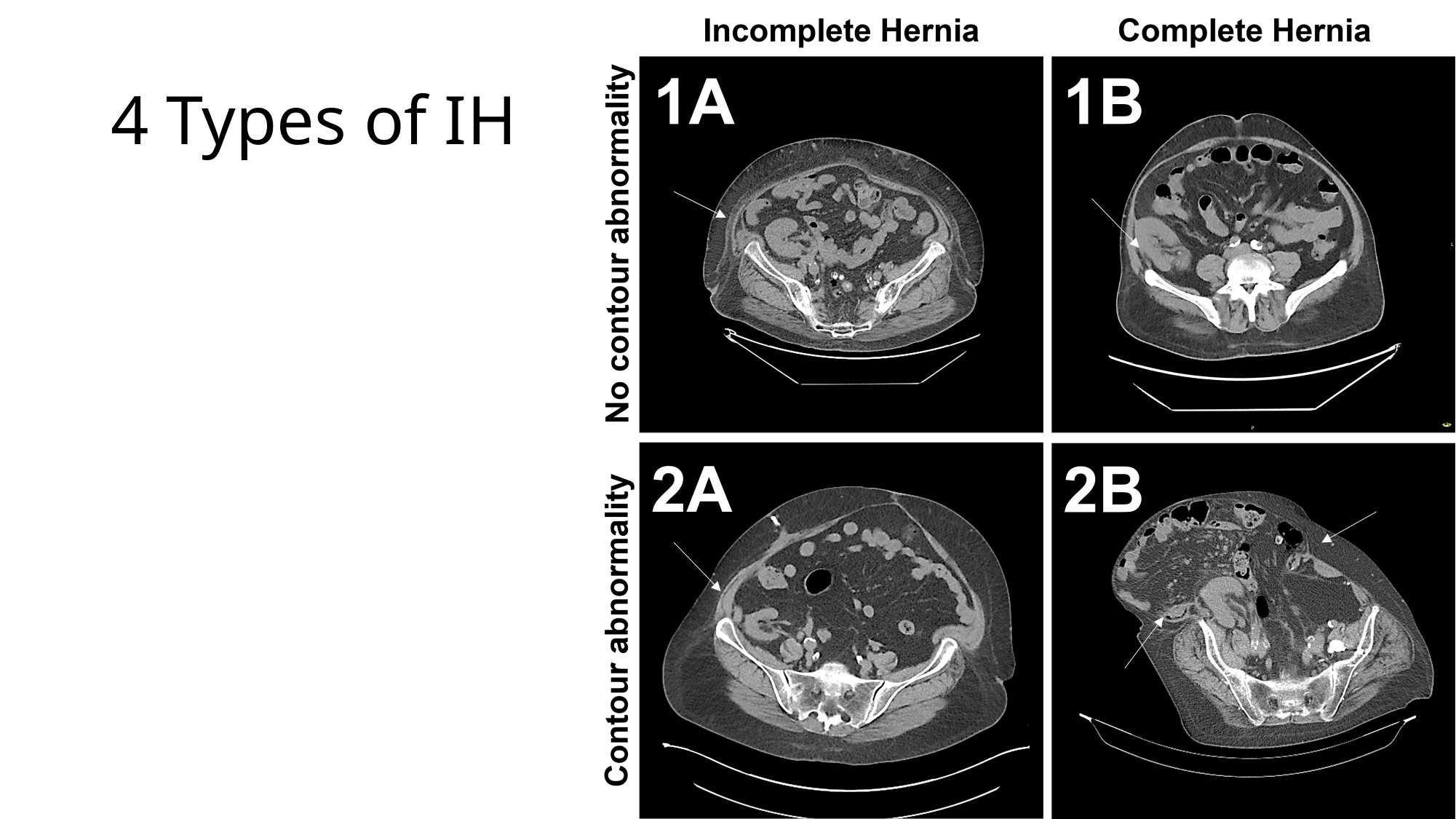

# 4 Types of IH
